# Dual Classification Approach for the Rapid Discrimination of Metabolic Syndrome by FTIR

**DOI:** 10.3390/bios13010015

**Published:** 2022-12-23

**Authors:** Kateryna Tkachenko, Isabel Esteban-Díez, José M. González-Sáiz, Patricia Pérez-Matute, Consuelo Pizarro

**Affiliations:** 1 Department of Chemistry, University of La Rioja, 26006 Logroño, Spain; 2 Infectious Diseases, Microbiota and Metabolism Unit, Infectious Diseases Department, Center for Biomedical Research of La Rioja (CIBIR), 26006 Logroño, Spain

**Keywords:** metabolic syndrome, infrared spectroscopy, point of care, metabolic signatures, chemometrics, classification strategy, health and wellbeing monitoring

## Abstract

Metabolic syndrome is a complex of interrelated risk factors for cardiovascular disease and diabetes. Thus, new point-of-care diagnostic tools are essential for unambiguously distinguishing MetS patients, providing results in rapid time. Herein, we evaluated the potential of Fourier transform infrared spectroscopy combined with chemometric tools to detect spectra markers indicative of metabolic syndrome. Around 105 plasma samples were collected and divided into two groups according to the presence of at least three of the five clinical parameters used for MetS diagnosis. A dual classification approach was studied based on selecting the most important spectral variable and classification methods, linear discriminant analysis (LDA) and SIMCA class modelling, respectively. The same classification methods were applied to measured clinical parameters at our disposal. Thus, the classification’s performance on reduced spectra fingerprints and measured clinical parameters were compared. Both approaches achieved excellent discrimination results among groups, providing almost 100% accuracy. Nevertheless, SIMCA class modelling showed higher classification performance between MetS and no MetS for IR-reduced variables compared to clinical variables. We finally discuss the potential of this method to be used as a supportive diagnostic or screening tool in clinical routines.

## 1. Introduction

The high prevalence of non-communicable diseases (NCD) in adults is reflected in increased costs for public health systems worldwide [1]. Among these NCD, metabolic syndrome (MetS) plays a significant role. MetS is often associated with an increased risk of diabetes and cardiovascular disease, resulting in increased incidence of morbidity and mortality and reduced quality of life [2,3,4,5,6]. Thus, the commensurate prevalence of metabolic syndrome burdens national health expenditure, representing a significant socio-economic problem, particularly in low- and middle-income countries [7,8,9,10]. However, MetS is a multifactorial disorder accompanied by conflicting opinions on its definition [11,12,13]. In particular, many different definitions have been proposed to describe MetS in adults. The main discrepancies were associated with inclusion and exclusion criteria adopted according to the World Health Organization (WHO), National Cholesterol Education Program (NCEP), Adult Treatment Panel III (ATPIII), and International Diabetes Federation (IDF). Finally, in 2009, the definition for metabolic syndrome was harmonised [14]: MetS is a disease formed by metabolic and vascular abnormalities, namely insulin resistance (IR), visceral adiposity, atherogenic dyslipidaemia, and oxidative and endothelial dysfunction. These risk factors easily predispose hyperglycaemia and hypertension, atherosclerotic vascular diseases and viral infection [15,16,17,18].

Given the complex and intertwined nature of MetS, it would be utopian to think that a single biomarker could define it unambiguously. Thus, parameters concerned around central obesity (waist circumference (WC)), hypertension (blood pressure), atherogenic dyslipidaemia (small low-density lipoprotein (LDL) and levels of high-density lipoprotein (HDL) cholesterol), and insulin resistance (fasting glucose levels) are usually measured to evaluate MetS diagnosis [19]. Due to the heterogeneity of these factors, people affected by metabolic syndrome are three times more likely to suffer acute myocardial infarction, cerebrovascular events, diabetes, or stroke. In addition, they have higher mortality rates [20]. Besides the economic impact, misdiagnosis or tardive diagnosis could lead not only to inefficient treatment outcomes but even to significant dysfunctions such as cancer [21,22]. Thus, early and proper diagnosis plays a crucial role in delaying the pathology’s onset or progression as much as possible and improving a patient’s condition.

Today, MetS diagnosis is based on several steps such as measuring metabolic markers of insulin resistance and other indices of metabolic syndrome (triglycerides, HDL cholesterol levels, and blood glucose) that are obtainable from routine clinical biochemistry laboratories, whereas blood pressure is measured in primary care [23]. The collection and analysis of samples also entails a waiting time for laboratory results and additional time for a new medical consultation. Although the proposed definition of MetS shares some common features, the clinical diagnosis lacks standardisation. On that basis, it was proposed that individuals showing a combination of any three out of these five simple clinical criteria were likely to be characterised by insulin resistance. Prospective analyses have also shown that any combination of these factors was predictive of an increased risk of both type 2 diabetes and cardiovascular disease. First, it is still challenging to identify a unified criteria for MetS applicable across all ethnicities. In addition, the contribution of each parameter seems to have different importance based on the evaluation adopted in each clinical environment (e.g., diagnosis focussed on glucose tolerance instead of obesity cut-offs). Moreover, there is variation in the cut-off values of diagnostic inclusion criteria (≥140/90 mmHg according to WHO vs. ≥130/85 mmHg according to ATP III for blood pressure). The application of MetS diagnosis in clinical practice could also be compromised, since most patient registries have missing data, limiting a study’s accuracy or leading to false-positive results. In addition, measurements such as WC, one of the predominant parameters for defining MetS, are not always feasible in patients because the diagnosis can often be limited by the patient’s inability to perform a complete physical examination.

Given these perspectives, the need for standardised clinical diagnostic tools and protocols becomes imperative in the prevention and diagnosis of MetS. For this reason, analysing global metabolic profiles instead of disparate clinical measurements could be essential in shedding light on MetS disarrangements. A multifactorial and complex pathology such as MetS seems to require an approach from a holistic functional perspective, so an analysis of metabolic profiles reflecting the global clinical status of a patient could represent a suitable alternative.

By now, metabolomics plays a key role as a powerful analytical tool that has been widely applied to investigate plenty of disorders and disarrangements [24,25,26]. Metabolomics analysis has the potential to discover biomarkers and allow for the detection of a wide range of metabolites. In recent years, there has been a great interest in extracting biomarkers from biofluids and, considering that blood is a biofluid containing numerous valuable metabolic information, it seems that it in particular, it appropriately reflects metabolic changes and disarrangements during disease initiation or progression [27,28]. In this context, techniques based on vibrational spectroscopy are particularly suitable as sample preparation is simple, non-invasive, rapid, and low-cost [29]. Therefore, the Fourier transformed infrared spectroscopy (FTIR) technique has been established as a reliable analytical tool in metabolomic-based studies [30,31,32,33,34]. Moreover, another significant advantage resides in the fact that FTIR is ideally suitable for acquose matrices such as blood [35,36]; the instrument requires the collection of only one blood sample, with little or almost null pre-treatment. In this study, we proposed an FTIR-based method that investigates many components at a time, which are registered as spectral signatures. The development of a chemometric strategy capable of extrapolating the most significant infrared (IR) signatures plays a crucial role in this study, since each spectrum is unique for every patient and reflects their metabolic status. Non-targeted metabolomic studies, such as the one presented here, aim to extract the metabolic signatures instead of individual biomarkers with limited potential, and this permits the classification of patients according to their molecular patterns, reflecting clinical/pathological conditions such as MetS or no MetS.

This method could greatly support clinicians, capturing the complexity of the MetS metabolic profile when the clinical indicators are missing or lacking sufficient discriminative power, revealing the globality of physiological disturbances. We do not want to underestimate the importance of clinical diagnosis at any time. Still, our main aim is to propose an alternative analytical strategy that could be of great diagnostic relevance and support, limiting the time and cost of clinical measurements.

## 2. Materials and Methods

### 2.1. Study Population

A total of 105 plasma samples from anonymous donors were recruited from Infectious Disease Area, Center for Biomedical Research of La Rioja (Logroño, Spain). This study was approved by the Committee for Ethics in Drug Research in La Rioja (CEImLAR) (23 April 2013, reference number 121) and a written informed consent was achieved from all participants. The patients were evaluated by the NCEP-ATP-III scale and, if eligible, were assigned to a metabolic syndrome category. MetS was defined as the concomitant presence of at least three of the following risk factors: elevated TGL (≥150 mg/dL), low concentrations of the fraction HDL cholesterol (<50 levels mg/dL in women or <40 mg/dL levels in men), increased WC (≥88 cm in women or ≥102 cm in men), elevated blood pressure (>130/85 mmHg), and elevated fasting glucose (>110 mg/dL or diabetes) [37]. Thus, the patients were divided into two groups by the criteria of MetS: 19 patients tested as MetS positive and 86 as MetS negative. The patients enrolled in this study were also characterised by the presence of viral load through serological evidence of HIV or co-infection of HIV/HCV. A correct distribution between patients with and without infection in both categories has been ensured to not introduce bias in future models developed for diagnosing MetS.

### 2.2. Sample Collection

Once drawn, the venous blood samples were centrifuged at 2200× *g* for 15 min at 4 °C and the obtained plasma were transferred into a clean Eppendorf tube. Aliquots of 200 μL of each sample were stored at −80 °C until the day of the analysis. Before FTIR measurements, plasma samples were defrosted during the night according to the optimised ultrasound-based protocol for lipidomic analyses developed in our research group [38].

### 2.3. Method

FTIR spectroscopy measurements were performed by a Spectrum-One ABB Miracle Type MB3000 FT-IR Spectrophotometer using a PerkinElmer liquid cell (Omni Cell, Specac Ltd., Orpington, UK) with CaF_2_ windows separated with a 50 μm Mylar spacer. The spectra from 25 μL of each plasma sample were recorded in the mid-IR region (4000–300 cm^−1^) in triplicate. A mean spectrum was subsequently obtained from the replicates recorded for each plasma sample. The sample temperature was maintained at 23.0 ± 1.0 °C, and a constant N_2_ purge was applied for atmospheric water vapour and CO_2_ suppression. A resolution of 2 cm^−1^ was obtained using 32 scans. In order to monitor the stability and reproducibility of the analytical system, quality control (QC) samples were processed similarly to the actual samples and inserted regularly. In addition, the instrument performance was verified at the beginning of each day of data collection using PE-specific reference standards.

### 2.4. Data Analysis

After data acquisition, the processing and computational analysis of raw metabolic data was performed using Unscrambler (version X 11.0, Camo ASA, Oslo, Norway), V-Parvus (version PARVUS2011, Michele Forina, Genoa, Italy), and Matlab (MATLAB 9.4 R2018a). Two different regions of the mid-IR spectrum were analysed: the first region examined was the biochemical “fingerprint region” at 1500–1050 cm^−1^, and the second was a higher region at 2950–2700 cm^−1^. Remaining wavenumber ranges, as they were affected by signal saturation effects caused mainly by strong water absorptions or noise, were removed, and not considered for further analysis. Given the high dimensionality of biological spectral data, many disturbing factors influence the spectral data acquisition, such as random noise, baseline distortions, or light scattering. Thus, the pre-processing step is imperative in analysis to reduce these factors. To compensate for instrumental artefacts and sample to sample variations, different pre-processing methods were evaluated individually or in combination to minimise the adulterant-unrelated variability, namely derivatives (e.g., Savitzky–Golay (S–G) first and second derivatives), standard normal variate (SNV), and extended multiplicative scatter correction (EMSC). Thus, better resolution of overlapping peaks and decreased scatter effects were ensured after applying the combination (S–G) smoothing and SNV.

The entire data set was split into two independent subsets to develop and validate the classifications proposed: a training set with 95 samples (used to optimise and develop the classification rules and models) and a test set with ten samples (never used in the construction of the classification but to evaluate their actual predictive ability). The test set used was the same for all methods applied and classifications developed. As a result, the smoothed and normalised output tables were always centred before additional multivariate analysis and classification algorithms.

## 3. Results and Discussion

After careful pre-processing, FTIR measurements were submitted for further multivariate analysis. Thus, five measured clinical variables and a total of 838 spectra variables over the wavelength ranges of 1583–1050 cm^−1^ and 2973–2700 cm^−1^ collected from 105 patients were included. The two main categories of this study were patients with and without metabolic syndrome, i.e., MetS and no MetS, respectively.

### 3.1. Descriptive Statistics

**Figure 1:**
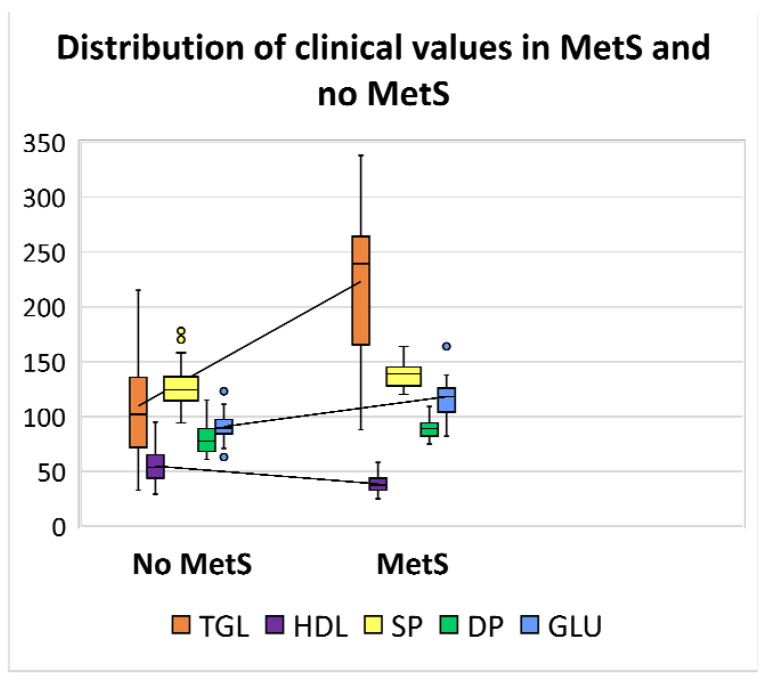
Box and whisker plot showing the distribution of clinical values levels in patients with MetS and no MetS. The line located in the middle of the box represents the median and is used to better visualise the differences between clinical parameters: triglycerides (TGL) levels are displayed in orange (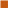); high density lipoprotein (HDL) in violet (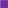); systolic pressure (SP) in yellow (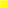); diastolic pressure (DP) in green (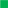); and glucose (GLU) in blue (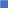).

Herein, an analysis was performed based on the distribution of five clinical parameters. It should be noted that one of the most critical clinical measurements, waist circumference, was not included in this study because most patients had missing data in the clinical register. Therefore, only parameters that were available for all patients have been used for the further comparative classification step. Thus, the descriptive statistics were calculated to analyse the distribution of clinical data in a box and whisker plot (Figure 1). The plot shows that TGL values seem to have more influence and variability between the two categories of patients; indeed, MetS patients have significantly higher values ranging from a minimum of 33 to 338 (mg/dL). The general distribution trend indicates that MetS patients also have slightly higher diastolic and systolic blood pressure values and glucose levels, whereas HDL values are lower, ranging from 25 to 95 (mg/dL). Table 1 shows the ranges of the collected values with the respective medians between the two categories.

### 3.2. Exploratory Analysis with PCA

An unsupervised pattern recognition method based on principal component analysis (PCA) was performed for the initial data overview and to investigate any possible clustering of samples based on five collected clinical parameters and 838 spectral variables, respectively.

**Figure 2:**
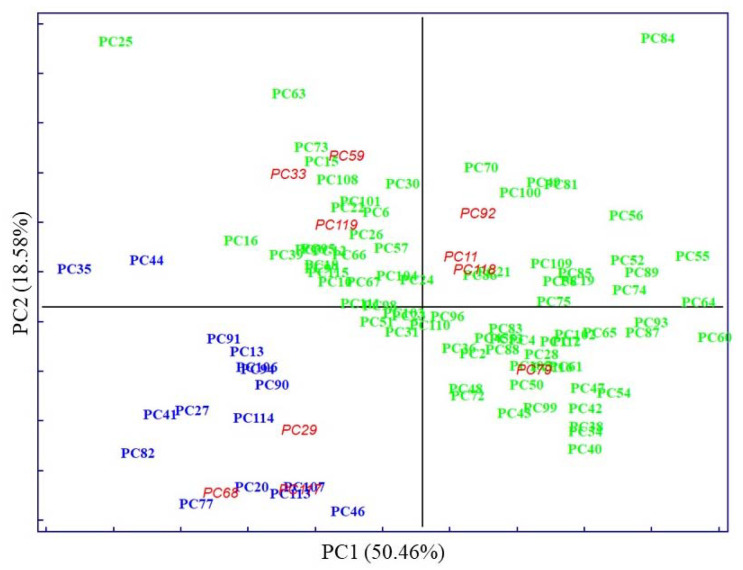
Scores for the plasma samples on the first two principal components explaining the variability in the dataset of five measured clinal parameters. The samples are labelled according to their specific pathology: no MetS (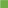), MetS (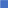), and external test samples (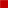).

**Figure 3:**
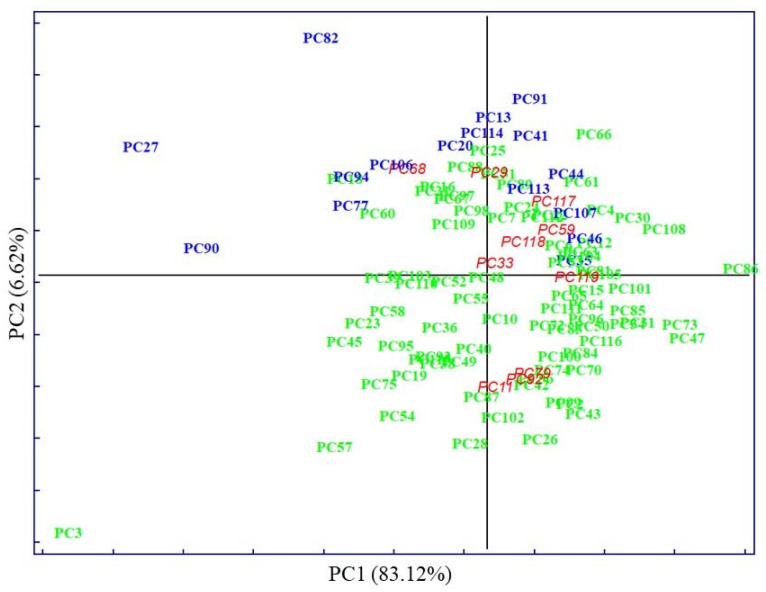
Scores for the plasma samples on the first two principal components explaining the variability in the IR spectral dataset. The samples are labelled according to their specific pathology: no MetS (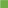), MetS (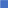), and external test samples (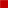).

The PCA score plot of clinical parameters, with 50.46% of explained variance by PC1, displays evident clustering according to known categories, delimitated by the parallel to the bisector of the second quadrant (Figure 2). Whereas PCA performed on pre-treated IR spectra accounted for 83.12% of explained variability on the PC1, evidenced by very subtle clustering between known categories (Figure 3).

In both cases, the first PCs explained most of the data’s variability. The distribution of samples in principal component space suggests that it only seems possible to address subsequent, direct discrimination in the case of analysis of clinical parameters. Thus, parameters such as TGL and GLU majorly contributed to the segregation of no MetS from MetS and the values of HDL contributed to the separation of MetS from no MetS, as was shown in preliminary analysis by descriptive statistics. No evident clustering among the two main categories was observed performing PCA on spectral variables; only a few outliers were determined and excluded from further analysis. The high degree of overlapping features among the two classes was expected, as most blood components are common in all individuals. This also indicates the need to perform a selection of relevant spectral variables, closely related to clinicopathological parameters of prognostic importance in MetS. Therefore, other chemometric strategies were used to investigate and highlight metabolomic differences in metabolic syndrome using IR spectra.

### 3.3. Supervised Techniques

The selection of variables in tandem with classification methods to extract reduced IR fingerprints that reflect the metabolic profiles of patients for a potential MetS diagnosis was studied. Therefore, a dual approach was applied based on a classification method on the one hand and a class modelling method on the other.

For its part, discriminant techniques focus on the differences between samples belonging to different categories, dividing the multidimensional space into as many subregions as the number of the considered classes. As a result of this work principle, every tested sample would always be assigned to one of the predefined categories, even in the case where an analysed sample truly belongs to a class not considered in the study. Regarding the above, it makes good sense to evaluate the application of a discriminant classification strategy in a two-class (binary) classification problem such as the one addressed in this paper. In particular, linear discriminant analysis (LDA), the most widely used classification algorithm, was used.

On the other hand, in contrast to class discrimination, class modelling approaches exploit similarities among inter-category samples to construct an individual model for every class independently from the others. Consequently, the developed class models may not entirely cover the original multivariate space. This fact opens the door to different assignment scenarios depending on whether a sample falls clearly into a single class region (so that it is assigned to that) or if it falls in overlapping regions (leading to a confusing classification in multiple classes), and, finally, when a sample falls outside every class model constructed (predicted as member of none of the considered categories). Therefore, due to their specific properties, modelling techniques, such as soft independent modelling by class analogy (SIMCA), are suitable for classification problems in which the emphasis is placed on a particular class of interest, as may be the case here with the MetS category.

#### 3.3.1. SELECT

Considering that IR data presents high dimensionality, eliminating the futile features due to noise and identifying the relevant and important variables to be applied in the following classification steps was imperative. Thus, the stepwise orthogonalization of predictors (SELECT) algorithm [39,40] was prioritised among other variable selection techniques since it enabled us to optimise discrimination by simultaneously performing feature selection and classification. Moreover, thanks to its stepwise decorrelation procedure, SELECT also avoids the presence of redundant information in the subset of selected significant predictors. In addition, it has previously demonstrated its accurate prediction ability in selecting the most important variable for the discrimination of pathological status [41,42]. Thus, SELECT was applied to extract the most significant wavenumbers from the IR dataset, providing input features for a further dual-classification approach. Based on the commonly established rule, the number of training objects selected was always at least three times greater than the number of finally selected wavenumbers. An in-depth study of the literature is encouraged to understand the algorithm’s rules [43].

#### 3.3.2. LDA on Clinical Parameters

LDA is a well-known and extensively applied powerful supervised chemometric classification technique [44]. Based on LDA classification rules, the objects are always classified in one of the predefined classes.

**Figure 4:**
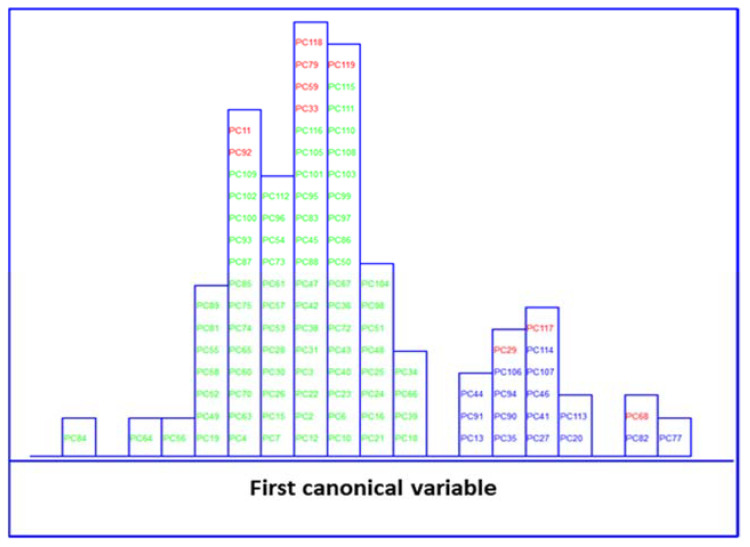
Histogram of the first canonical variable for the discrimination of MetS (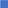) and no MetS (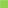) patients within included (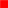) test set, after performing LDA in the stratification approach based on clinical parameters (*y*-axis indicates the maximum discrimination power between categories).

LDA of five clinical parameters, built by leave one out (LOO) cross-validation, was performed to evaluate the feasibility of this classification methodology to differentiate between MetS and no MetS patients. Excellent discrimination among categories was achieved, providing a 100% level of correctly classified samples for no MetS subjects and patients with metabolic syndrome, respectively. Satisfactory external prediction performances ranging from 98.73% to 100% were achieved for both categories (within one no MetS subject classified as MetS), respectively (Table 2). Furthermore, a clear interclass separation achieved between these main categories can also be visually appreciated in the corresponding discriminative histogram (Figure 4). This classification performance was almost predictable since the PCA results already showed a clear clustering between the two groups.

The object belonging to the category MetS which was classified as no MetS was characterised by the following clinical parameters: 213 mg/mL of TGL, 76 mg/mL of HDL, 139 mmHg of SP, 83 mmHg of DP, and 102 mg/mL of GLU. As we can see, two out of five parameters have increased values, and the DP parameter is very close to the cut-off value, which is 85 mmHg based on the NCEP-ATP-III scale. Thus, this patient might instead be classified as MetS positive, presenting almost three out of five clinical parameters with augmented values. In addition, as we said above, the TGL parameter has a major contribution, among other parameters, to MetS classification. Thus, the plausible explanation could be that this subject, who has greater values of TGL, is more likely to be classified as MetS by LDA rather than no MetS. However, as we highlighted before, the eligibility criteria can be very insidious and create confusion and misassignment, worsening and delaying the patients’ well-being.

#### 3.3.3. SELECT-LDA on IR Wavenumbers

**Figure 5:**
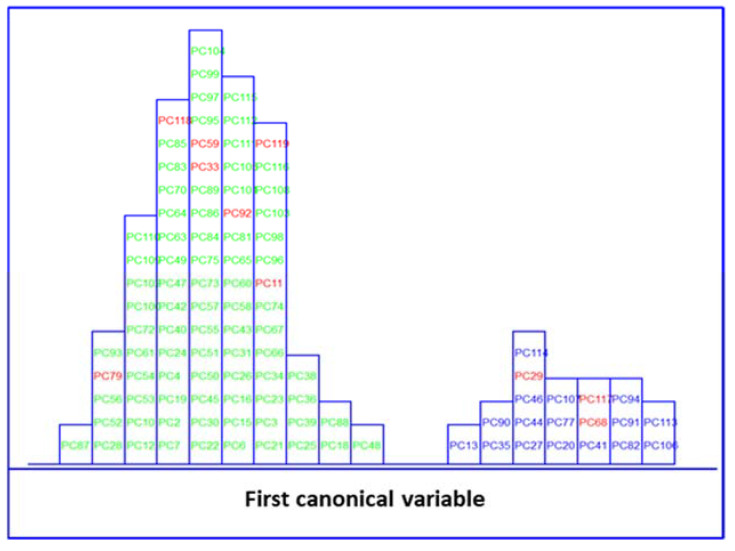
Histogram of the first canonical variable for the discrimination of MetS (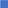) and no MetS (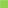) patients within the included (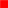) test set, after performing SELECT-LDA in the stratification approach based on 20 IR variables (*y*-axis indicates the maximum discrimination power between categories).

Likewise, LDA on the IR dataset, containing 838 wavenumbers, was also performed. Before LDA analysis, as explained above, SELECT was applied to extract those predictor variables correlated with the discrimination between categories here considered. Therefore, based on the SELECT rules, 20 selected spectra variables were decorrelated from other signals and used for LDA. The 20 selected features showed an outstanding classification performance and the results were higher in performance than LDA results on clinical parameters, achieving 100% in classification and external prediction, respectively. The results of the SELECT LDA performance are displayed in Table 3. The suitability of the classification strategy applied to reduced IR plasma signatures can be visually appreciated in Figure 5. A discriminative histogram shows a clear group separation on the first canonical variable.

#### 3.3.4. SIMCA

In an attempt to go one step further in this classification strategy, it was decided to build optimised class models based on clinical parameters and the subset of reduced IR signatures selected by SELECT. SIMCA often outperforms other classification methods, where a new sample will always be classified in one of the predefined categories. Classification methods such as LDA are based on the development of classification rules and delimiters between classes, whereas in class models, significance limits are built for the specified classes. These limits define the membership parameters for each class; thus, an unknown sample can be classified as not belonging to any defined categories because it is not included in any of its class spaces. SIMCA class modelling uses the number of true/false positives and negatives and statistics, showing the ability of a classification model to recognise class members (*sensitivity* or true positive rate) and showing how good the model is for identifying strangers (*specificity* or true negative rate). Moreover, SIMCA class modelling is often used to describe the class structure of the data set, requiring little or no prior assumptions to build the model.

On applying SIMCA, independent PCA modelling is performed for each class; each sample is fitted in a PCA model to check the separation between classes [45]. This model uses the optimal number of principal components that best describes and groups an individual class. This model can then be used to classify new samples whose class is unknown. The principal components are obtained usually using the NIPALS (non-iterative partial least squares) algorithm after separate autoscaling of the data. Finally, the models built for the different classes are compared by studying their differences and analogies [46]. Each class is modelled independently; thus, it is sensitive to the quality of the data used to generate the principal component models for each class in the training set (at a 5% significance level).

##### SIMCA on Clinical Parameters

**Figure 6:**
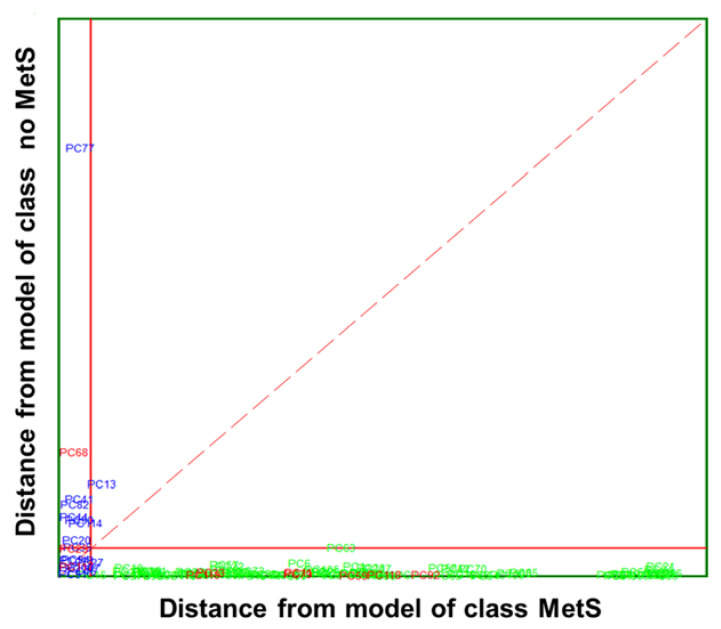
Cooman’s plot displaying the results obtained by applying SIMCA class-modelling to clinical parameters: MetS (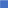) and no MetS (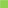) patients within the included (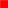) test set. The red solid line indicates a confidence level for class space at 95%. The red dashed line indicates equal class distance.

Herein, SIMCA modelling was performed on five clinical parameters (Table 4). A class modelling of five clinical parameters of MetS was built using 4PCs for the inner space of classes, achieving satisfactory results in both internal prediction (LOO) and external prediction 98.95%. SIMCA builds a mathematical model of the category with its principal components and a sample is accepted by the specific category if its distance to the model is not significantly different from the class residual standard deviation. The results of SIMCA modelling can be visually appreciated by a Cooman’s Plot, representing the samples’ distances against each of the two models. The Cooman’s plots were built considering a 95% confidence level to define the class space and the unweighted augmented distance. This diagram is an effective visual representation that directly indicates the quality of the model constructed with the magnitude of the distance between categories. Thus, the distances to the principal component models and SIMCA approximation in a two-class problem for the class of MetS and no MetS are plotted in Figure 6. No clear outliers were observed, but several samples that fall into the joint space of both categories belong mainly to the MetS category. This relatively large number of samples plotted in the class-space common (overlapping) to the two models representing MetS and no MetS patients, as well as the considerable amount of no MetS samples located near their class boundary, suggest potential specificity problems associated with this classification approach based on clinical parameters. Therefore, the distribution of some samples from the MetS category in the area of relative indecision (small left quadrant) could be due to the unequivocal diagnostic parameters defining metabolic syndrome. In fact, these patients have three out of five altered parameters not necessarily similar. In addition, some parameters may be much less marked than others, confounding the decision about their location inside the model.

The data modelling power (MP) and discriminatory power (DP) of the SIMCA class modelling of clinical parameters are presented in Table 4. The MP describes how well a variable helps each principal component to model variation in the data, and discriminatory power (DP) describes how well a variable helps each principal component model to classify samples in a training set. The first detail that can be noticed is that, comparably, the MP in no MetS is consistently higher for all parameter pairs. This was expected as the distribution of the values of clinical parameters for each class of patients was significantly different. Nevertheless, the values of TGL have the highest modelling power in both MetS and no MetS categories, with values of 0.94 and 0.96, respectively. This ability of TGL to discriminate between the two groups is justified by previous studies, as metabolic syndrome patients should have significantly higher TGL values. This difference in modelling power is especially remarkable by the measured glucose (0.97 vs. 0.84) and HDL (0.94 vs. 0.79). In addition, clinical parameters such as glucose and HDL also showed significant discriminant power, with values of 2.63 and 2.58, respectively. These two parameters are also perfectly in line with the data collected from our patients. The MetS group is characterised by high glucose and low HDL values. These same parameters are often responsible for the presence or future development of comorbidities in patients such as diabetes, cardiac disease, and obesity. Other clinical parameters seem to contribute less to the principal component models; indeed, no significant difference was observed in the values distribution of SP or DP between the two categories.

##### SELECT-SIMCA on IR Wavenumbers

The best recognition ability (percentage of the samples in training set correctly classified during the modelling step) afforded by SIMCA was achieved by only ten of 20 previously selected wavenumbers by SELECT, providing 98.94% in classification and 95.79% in external prediction, respectively. Interestingly, eight out of ten selected wavenumbers belong to the ‘’fingerprint region’’, which reflects the production of characteristic perturbations in the metabolome and other such variations. The absorption pattern in this area is highly complex; that same inherent complexity makes it unique for each sample and reflects its pathophysiological status. Thus, eight of the selected IR spectral wavenumbers may reflect the current status of the organism and could be directly correlated with the presence or absence of the disease. The results of SIMCA performance applied to clinical variables and to reduced number of IR spectral variables are summarised in Table 5.

**Figure 7:**
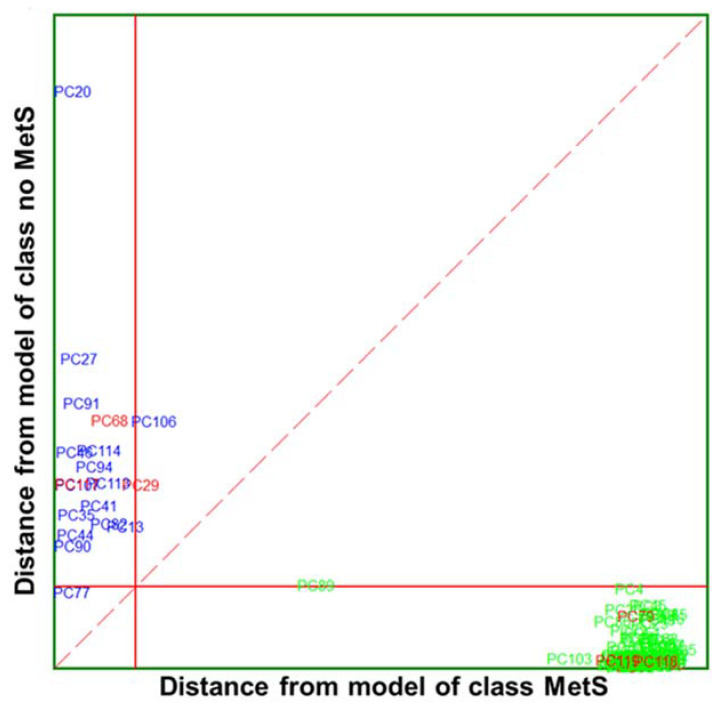
Cooman’s plot displaying the results obtained by applying the SELECT-SIMCA class-modelling to ten selected IR signals: MetS (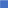) and no MetS (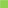) patients within included (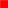) test set. The red solid line indicates a confidence level for class space at 95%. The red dashed line indicates equal class distance.

A Cooman’s plot is presented to show discrimination between the two MetS categories of IR variables (Figure 7), where the distance to the PC models for MetS and no MetS are displayed. Compared to the Cooman’s plot of clinical parameters, it is observed that there is better separation and discrimination between categories. The Cooman’s plot showed a high degree of interclass specificity and a patently clear separation between class models, with a significant improvement from the models constructed from available clinical parameters to those constructed from IR variables. The no MetS patients appear evidently segregated and concentrated forming a dense cluster at large distances from the model of MetS class. Likewise, the vast majority of MetS samples fall clearly and univocally into their class region, far from the class limit for the no MetS model. Furthermore, the single MetS sample located in the inconclusive classification region is virtually placed above the membership threshold.

From ten selected wavenumbers, the highest discriminant power (5.87) was obtained by the 1133.09 cm^−1^ spectra variable from the ‘’fingerprint region’’ (Table 6), followed by 4.31 for 1557.40 cm^−1^ and 4.29 for 2948.94 cm^−1^ from the higher spectral region. The average discriminant power for IR variables is higher compared to DP values obtained with SIMCA modelling of clinical parameters, indicating the increased suitability of the method compared to those using values obtained from clinical measurements. Likewise, the contribution of IR variables to the model variation was of major strength compared to clinical parameters. Thus, all the selected variables contributed equally to marking the difference between MetS and no MetS with an MP equal to 1.00. Furthermore, the distance between classes was 5.19, significantly higher than in the case of SIMCA class modelling applied to clinical parameters (4.26). These results highlight that the proposed method outperformed in accuracy and specificity of the evaluation parameters used in clinical practice. Since the clinical diagnosis of metabolic syndrome lacks standardisation, the results of the obtained model capacity could greatly support clinical decisions, for example, in terms of exclusion and inclusion evaluation criteria for MetS discrimination.

Our principal aim was to obtain optimal segregation between patients without additional clinical, physical, or ethnic data, and this goal was achieved.

#### 3.3.5. Biochemical Reasoning of Ten Extracted Signals

Herein, we presented a simple, non-invasive, low-cost FTIR-based method for rapid discrimination between MetS and no MetS patients. The use of FTIR spectroscopy is gaining momentum for diagnosis of multiple disorders, from infectious diseases such as hepatitis C and B viruses or malaria to cancers [47,48,49,50,51,52,53]. Due to its ease of use and portability, the potential for using FTIR techniques in clinical environments is within reach. Our strategy extracted the metabolic signatures, instead of individual biomarkers with limited potential, that permit the classification of patients according to molecular patterns. Thus, the FTIR technique provided an overview of spectral changes associated with lipid, protein, or carbohydrate metabolisms.

Ten out of twenty previously selected wavenumbers showed higher discriminant power than clinical parameters. Thus, among these, influential bands at 1578.61, 1562.22, and 1557.40 cm^−1^ could be assigned to [δ (N-H) + ν (C-H)] of the amide II region of proteins. These discriminative signals may suggest some link with HDL lipoproteins, which showed significant influence among five clinical factors for the classification of MetS and no MetS subjects. Likewise, the higher absorbance in peaks at 2860.22 cm^−1^ and 2948.94 cm^−1^ could be attributed to CH3 and CH2 sym. stretching of lipids or carbohydrates, which is perfectly congruent with the formulated theories about MetS impairments and their possible implication in the disease. Moreover, as discussed above, TGL and GLU levels seemed to have more influence and variability between the two categories of patients; thus, these attempted assignments properly reflect the actual situation of the patient’s metabolism. In addition, the variable at 1133.09 cm^−1^ could be associated with stretching C-O/C-O(H) of carbohydrates or proteins, since it was already shown that the parameters such as glucose or HDL have remarkable modelling and discriminant powers compared to other measured factors.

In this study, the selected spectral biomarkers perfectly reflect the clinical reality of the patient’s metabolic profile. Thus, the explanation of the most significant spectral bands confirms the potential of FTIR spectroscopy to deal with such a complex disorder as MetS.

## 4. Conclusions

We firmly believe that this alternative analytical strategy could be of great diagnostic relevance and support for clinicians, limiting the time and cost of MetS diagnosis. Moreover, the evaluation of the metabolic profile captures the globality of physiological disturbances, whereas clinical indicators often lack sufficient discriminative power. The results indicate the possibility of rapid application of this strategy to screen for patients with metabolic syndrome. The LDA classifications and SIMCA developed models demonstrated that the spectral variables could provide the same discriminative results as measured clinical parameters. Therefore, why take five measurements when one measurement could provide the same classification ability, greatly stratifying categories of patients? The proposed FTIR method is quick, simple, and non-invasive, and it could be perfectly implemented for large scale-analysis in clinical routines. The principal limitation of this study resides in the relatively tiny sample size at our disposal. In addition, this is a cross-sectional study; therefore, no data on confounding factors (such as gender, age, or diet) were routinely included. The results of a more extensive data set would be required to strengthen the validity of the adopted classification strategy and lead to a firmer conclusion.

## Figures and Tables

**Table 1:** The distribution of the clinically measured parameters in MetS and no MetS patients expressed in mg/dL and in mmHg.

Category		MetS		No MetS		
Clinical Parameters	Max	Min	Mean	Max	Min	Mean
Systolic blood pressure	174	120	136	178	94	126
Diastolic blood pressure	109	75	87	115	61	79
Triglycerides	338	88	242	215	33	109
HDL	58	25	37	95	29	55
Glucose	164	82	114	123	63	91

**Table 2:** Results of LDA classification performance on clinical parameters.

Clinical Parameters	Classification (%)	External Prediction (%)	Total Rate (%)
MetS	100	100	100
No MetS	100	98.73 (1)^1^	99.36
Total rate	100	98.94	99.47

^1^ The one corresponds to one misclassified subject in cross-validation.

**Table 3:** Results of SELECT LDA classification performance on 20 IR selected spectral variables.

Clinical Parameters	Classification (%)	External Prediction (%)	Total Rate (%)
MetS	100	100	100
No MetS	100	100	100
Total rate	100	100	100

**Table 4:** The values of discriminant and modelling powers of clinical parameters after SIMCA class-modelling.

Clinical Parameters	Discriminant Power	Modelling Power	
		Category MetS	Category No MetS
Systolic blood pressure	1.99	0.70	0.73
Diastolic blood pressure	2.01	0.70	0.73
Triglycerides	2.18	0.94	0.96
HDL	2.34	0.79	0.94
Glucose	2.36	0.84	0.97

**Table 5:** The results of SIMCA class-modelling performance on clinical parameters and ten selected IR spectral variables.

Variables	Classification (%)	LOO (%)	CV Efficiency (%)	Efficiency Forced Model (%)	Total Rate (%)
5 clinical measurements	98.59	97.18	87.05	95.68	100
10 IR selected wavenumbers	97.18	94.37	87.92	97.86	100

**Table 6:** Discriminative and modelling powers of ten selected spectra variables after SELECT-SIMCA class modelling.

Wavenumber (cm^−1^)	Discriminant Power	Modelling Power	
		Category MetS	Category No MetS
2860.22	3.77	1.00	1.00
1423.36	4.23		
1562.22	3.66		
1578.61	3.75		
1108.98	3.70		
1316.32	3.64		
2948.94	4.29		
1557.40	4.31		
1133.09	5.86		
1247.85	3.58		

## Data Availability

Not applicable.
